# Promoter methylation analysis of *DKK2* may be a potential biomarker for early detection of cervical cancer

**DOI:** 10.2478/abm-2022-0022

**Published:** 2022-08-31

**Authors:** Xian Zhang, Aihua Li, Jie Wu, Yu Wu, Xiaoping Ma, Yanjun Liu, Qingfa Chen, Yan Zhang

**Affiliations:** Department of Gynecology and Obstetrics, Liaocheng People's Hospital and Liaocheng Clinical School of Shandong First Medical University, Liaocheng, Shandong 252000, China; Department of Pathology, Affiliated Hospital of Qingdao University, Qingdao, Shandong 266000, China; Institute of Tissue Engineering and Regenerative Medicine, Liaocheng People's Hospital and Liaocheng Clinical School of Shandong First Medical University, Liaocheng, Shandong 252000, China

**Keywords:** biomarkers, data mining, DKK2 protein, human, DNA methylation, human papillomavirus, uterine cervical neoplasms

## Abstract

**Background:**

Dickkopf 2 (*DKK2*) plays an important role in multiple cancers. Its potential value in the clinical diagnosis of cervical cancer has remained unclear.

**Objectives:**

To investigate the expression and promoter methylation levels of *DKK2* in cervical cancer and their clinicopathological associations.

**Methods:**

We used the Gene Expression Omnibus, Oncomine, Cancer Genome Atlas, and University of ALabama at Birmingham CANcer data analysis databases, reverse transcription-PCR, and methylation-specific PCR analysis to predict and examine the expression of DKK2 mRNA and *DKK2* methylation levels in cell lines and cervical cancer tissues from 79 patients with cervical cancer and 63 with cervical precancerous lesions including 25 with low-grade squamous intraepithelial lesions (LSIL) and 38 patients with high-grade squamous intraepithelial lesions (HSIL).

**Results:**

DKK2 mRNA expression was downregulated in all cancer cell lines and cervical cancer tissues, whereas hypermethylation of *DKK2* was higher in cervical cancer tissue samples. *DKK2* methylation in cervical cancer was significantly higher than that in HSIL (χ^2^ = 8.346, *P* = 0.004), whereas *DKK2* methylation in HSIL was significantly higher than that in normal cervical samples (χ^2^ = 7.934, *P* = 0.005) and in LSIL samples (χ^2^ = 4.375, *P* = 0.037). *DKK2* silencing caused by its promoter hypermethylation was confirmed by treatment with the methyltransferase inhibitor 5-Aza-dC in cell lines. Patients with lymph node metastasis exhibited increased promoter methylation frequency (χ^2^ = 5.239, *P* = 0.022) and low DKK2 mRNA expression (χ^2^ = 3.958, *P* = 0.047) compared with patients with no lymph node metastasis. Patients with high-risk human papillomavirus infection exhibited increased promoter methylation frequency (χ^2^ = 6.279, *P* = 0.015).

**Conclusions:**

*DKK2* epigenetic changes of DKK2 may play a key role in the development of cervical cancer, suggesting that *DKK2* hypermethylation could be used as a triage test for screening, early diagnosis, or risk prediction of cervical cancer.

Cervical cancer is the most common gynecological malignancy and is especially prevalent in young women [1]. While cervical cancer is often curable if detected at an early stage, the overall survival in patients with advanced cervical cancer remains poor. Therefore, better treatments and molecular markers related to carcinogenesis and progression that can help clinical practice are needed urgently. Screening is key for the early detection of precancerous lesions and cancer. ThinPrep cytologic tests (TCT) and human papillomavirus (HPV) detection are 2 important and commonly used methods to screen for cervical cancer [2]. TCTs are inexpensive, but their performance depends on several subjective human factors, which can lead to misdiagnosis and missed diagnosis, and poor accuracy. The sensitivity for detecting HPV is high, but most cases of HPV infection are transient, leading to poor specificity. Cytological screening strategies combined with HPV testing have contributed to a substantial reduction in the incidence and mortality of cervical cancer [3]. However, overall, the positive predictive effects are moderate, as only a small subset of people with HPV will eventually develop cervical cancer. Gene methylation analysis is a nonmorphological molecular detection method, which can provide an objective and potential shunt method for high-risk HPV-positive women. Triage tests using DNA methylation may be helpful to differentiate women who are at high risk of rapidly developing cervical cancer from women with low risk.

DNA methylation of tumor-related genes is closely related to early tumor progression, in a cancer type specific manner, and may provide information that can aid early screening of cervical cancer [4]. Aberrant promoter methylation, and epigenetic changes in tumor suppressor genes, either inactivating or silencing, are associated with tumorigenesis and progression [5,6,7,8]. In humans, DNA methylation occurs almost exclusively at the carbon 5 position on cytosine residues in CpG dinucleotides, which are concentrated in distinct GC-rich regions called “CpG islands” (CGIs) [8]. CpG island hypermethylation of tumor suppressor genes has been linked to the development of numerous human cancers, including cervical cancer [9, 10]. The Wnt/β-catenin signaling pathway is known to be regulated by several secreted antagonists, such as members of the Dickkopf (DKK) protein family (DKK1, DKK2, DKK3, and DKK4), and has a function in embryonic development and tumorigenesis [11]. *DKK2*, located at 4q25, functions to produce an antagonist of canonical Wnt/β-catenin through its receptor low-density lipoprotein-receptor-related protein 5/6 (LRP5/6). The *DKK2* promoter has a typical CpG island and is, therefore, under epigenetic regulation through promoter CpG methylation. *DKK1* and *DKK2* epigenetic silencing through promoter methylation have been observed in multiple cancers, including colorectal cancer, breast cancer, Ewing sarcoma, and gastric cancer [12,13,14,15,16,17,18]. However, its precise cellular function in cancer remains elusive.

Although DKK2 plays a significant role in many tumors, its correlation with clinicopathological characteristics such as lymph node metastasis and HPV infection is still unknown in cervical cancer. Here, we evaluated the mRNA expression profiles of *DKK2* and its epigenetic alterations in cervical cancer cell lines and in samples from patients with cervical cancer.

## Methods

### Data mining and analyses

To compare gene expression differences between healthy donors and patients with cervical cancer, all clinicopathological data related to DKK2 expression profiles (GSE6791, GSE7803, GSE9750, and GSE7410) were carefully selected from the Gene Expression Omnibus (GEO) database, The Cancer Genome Atlas (TCGA) database (https://www.cancer.gov/about-nci/organization/ccg/research/structural-genomics/tcga), and Oncomine websites (https://www.oncomine.org/resource/login.html). GraphPad Prism was used to obtain the scatter diagram. *DKK2* methylation expression data was mined in University of ALabama at Birmingham CANcer (UALCAN) databases (http://ualcan.path.uab.edu/) [19, 20]. This study was conducted following the Reporting Recommendations for Tumor Marker Prognostic Studies (REMARK) checklist [21].

### Cell lines and clinical samples

Ect1/E6E7 (catalog No. AC39960) normal cervical epithelial cell and HPV-positive HeLa (catalog No. TCHu187), HPV-positive CaSki (catalog No. BNCC338223), HPV-negative HT-3 (catalog No. HTB-32), and HPV-negative C33A (catalog No. BNCC337882) cervical cancer cell lines were screened. Cells were purchased from American Type Culture Collection (ATCC) or BeNa Culture Collection (Beijing, China) and cultured in RPMI-1640 medium (Gibco).

We analyzed a series of cervical specimens with histological diagnoses collected between May 2016 and November 2017 at Liaocheng People's Hospital (Shandong, China). At the time of surgical resection or cervical biopsy, 79 invasive cervical cancer tissues and 63 cervical intraepithelial neoplasia (CIN) tissues were obtained. Of the CIN tissues, 25 were classified as low-grade CIN (low-grade squamous intraepithelial lesions, LSIL) and 38 as high-grade CIN (high-grade squamous intraepithelial lesions, HSIL), and the latter included both CIN2 and CIN3. Normal cervical tissue (29 cases) was retrieved from patients with uterine leiomyomas who underwent a hysterectomy. All samples were stored at −80 °C until analysis. Genotyping was used to detect HPV16 and HPV18 viral DNA [20]. All cases had a confirmed diagnosis, as well as confirmed HPV typing and histopathological type. Exclusion criteria included samples from patients undergoing chemotherapy or radiotherapy, immunocompromised patients, pregnancy, diagnosis of other cancers, uterine cervix operations, and chronic or acute viral infection. Documented written informed consent was provided by all patients for tissue sample collection. The present study was approved by the medical ethics committee of Liaocheng People's Hospital, China (No. 2016022), and was consistent with relevant national regulations and laws, including the People's Republic of China Regulations for the Management of Medical Institutions (promulgated by the Order No. 149 of the State Council on February 26, 1994; and revised in accordance with the Decision of the State Council on Amending Some Administrative Regulations on February 6, 2016), and international medical ethics documents, including the principles of the World Medical Association (WMA) Declaration of Helsinki and its contemporary amendments (2013).

### Reverse transcription-polymerase chain reaction

DKK2 mRNA expression was examined by reverse transcription-polymerase chain reaction (RT-PCR) analysis as previously described [18]. Total RNA was extracted from cell lines, CIN, normal cervical tissues, and tumor samples using TRIzol reagent (Invitrogen). *DKK2* expression level was calculated relative to that of glyceraldehyde-3-phosphate dehydrogenase (GAPDH) in each sample by RT-PCR. Primer pairs used for mRNA expression and the size of PCR products are described in **Table 1**. The products were separated in 2% agarose gels using a DL1000 marker set (catalog No. 3591A; Takara) marker set.

**Table 1:** Primer pairs used for mRNA expression and the size of PCR products

**Gene**	**Primer information (5′-3′)**	**Product size**
*DKK2*	5′-GTACCAAGGACTGGCATTCG-3′ (F) 5′-ATCTCGGTGGCAGCGCTTCT-3′ (R)	169 bp
*GAPDH*	5′-CCAGCAAGAGCACAAGAGGAA-3′ (F) 5′-CAAGGGGTCTACATGGCAACT-3′ (R)	114 bp
*DKK2* (M)	5′-AGAGTTAAATCGTCGAGATTTC-3′ (F) 5′-CTAAAAACAATCAAATACGAAACG-3′ (R)	146 bp
*DKK2* (U)	5′-GGAGAGTTAAATTGTTGAGATTTT-3′ (F) 5′-ACTAAAAACAATCAAATACAAAACA-3′ (R)	149 bp

*DKK2*, gene for Dickkopf 2; F, forward primer; *GAPDH*, gene for glyceraldehyde-3-phosphate dehydrogenase; M, methylated-specific primers; PCR, polymerase chain reaction; R, reverse primer; U, unmethylated-specific primers.

### DNA extraction and bisulfite modification

DNA samples were extracted from tissues and cell lines. Genomic DNA (1 μg) was bisulfite-modified and purified following the guidelines described by the manufacturer. After desulfonation with NaOH and precipitation with ethanol, the final products were dissolved in 20 μL of Tris-EDTA buffer and stored at −80 °C.

### Methylation-specific PCR

*DKK2* gene methylation was determined using a Methylation-Gold Kit (Zymo Research). Primer pairs used for methylation analysis and PCR size products are shown in **Table 1**, as previously described [12, 23]. Briefly, 5 min predenaturation occurred at 95 °C, 30 s denaturation for 35 cycles, 30 s annealing at 54 °C (unmethylated-specific PCR) or 58 °C methylation-specific PCR (MSP), and 10 min extension at 72 °C. As a positive control for methylation, DNA obtained from normal tissue was methylated in vitro with M.Sssl CpG methyltransferase (New England BioLabs). A water blank (no template DNA) was also included as a negative PCR control. The products were separated in 2% agarose gels using a DL1000 marker set (catalog No. 3591A; Takara) marker set.

### 5-Aza-dC treatment

HeLa, CaSki, HT-3, and C33A cells (2 × 105) were treated with 10 μmol 5-Aza-2′-deoxycytidine (5-Aza-dC) (Sigma) for 96 h. After adding the demethylation treatment, the medium was changed every 24 h. RNA and DNA were isolated according to routine procedures.

### Statistical analysis

SPSS Statistics for Windows (version 17.0) was used for all statistical analyses. Student *t* tests were performed for data mining, and χ^2^ or Fisher exact tests were performed for RT-PCR and MSP analyses. *P* < 0.05 was considered significant.

## Results

### DKK2 mRNA expression in cervical cancer cell lines and tumor specimens

**Figure 1:**
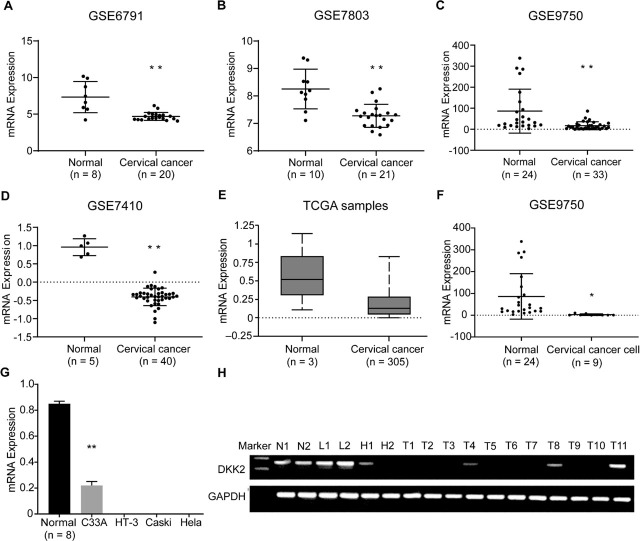
DKK2 mRNA expression in cervical cells and tissues. (**A–E**) RT-PCR analysis of DKK2 mRNA expression in cervical normal and cancer tissues. ^**^P < 0.01 compared with normal tissues. (**F**) RT-PCR analysis of DKK2 mRNA expression in normal cervical epithelial cell lines and in 9 human cervical cancer cell lines. ^*^P < 0.05 compared with normal cervical epithelial cell lines. (**G**) RT-PCR analysis of DKK2 mRNA relative expression in normal cervical epithelial cell lines and 4 human cervical cancer cell lines (Hela, Caski, HT-3, and C33A cell lines). ^**^P < 0.01 compared with normal cervical epithelial cell lines. (**H**) DKK2 mRNA expression in LSIL, HSIL, tumor, and normal cervical tissues. The PCR products were separated in 2% agarose gels using a DL1000 marker set (catalog No. 3591A; Takara) marker set; the band of interest is between markers 100 bp and 200 bp at 169 bp. DKK2, Dickkopf 2; GAPDH, glyceraldehyde-3-phosphate dehydrogenase; H, HSIL; L, LSIL; N, normal; RT-PCR, reverse transcription-polymerase chain reaction; T, tumor; TCGA, The Cancer Genome Atlas; bp, base pairs

We examined DKK2 mRNA expression in 79 normal cervical tissues (50 by data mining, 29 by RT-PCR), 9 cervical cancer lines (HT-3, C4-I, CaSki, MS751, C33A, SiHa, SW756, ME-180, and HeLa cell lines by data mining, HeLa, HT-3, C33A, and CaSki cell lines by RT-PCR), and 498 cervical cancer samples (419 by data mining, 79 by RT-PCR). Based on data from the GEO database (**Figures 1A–D**), DKK2 mRNA expression was lower in cervical cancer tissues compared with normal tissues (*P* < 0.0001 for **Figures 1A, B,** and **D**, *P* = 0.0004 for **Figure 1C**). However, based on data from the TCGA database (**Figure 1E**), the difference was not significant (*P* = 0.848), although the levels of DKK2 mRNA expression showed a similar trend to that of the GEO database. Compared with normal cervical epithelial cells, expression in cervical cancer cells including HT-3, C4-I, CaSki, MS751, C33A, SiHa, SW756, ME-180, and HeLa cervical cancer cell lines was lower, as shown in **Figure 1F** (*P* = 0.023).

We then examined DKK2 mRNA expression both in cervical cancer cell lines and in tissue samples. Our RT-PCR analysis indicated that DKK2 mRNA expression was weak in HPV-negative C33A cells, and silenced in HPV-negative HT-3 cells and HPV-positive CaSki and HeLa cells, as compared with the normal cervical epithelial cell line Ect1/E6E7 (**Figure 1G**). All 29 normal cervical tissues, and 46 of 63 (73.0%) CIN samples, showed DKK2 mRNA expression (**Table 2**). Among 79 cervical cancer samples analyzed, only 25 of 79 (31.6%) showed DKK2 mRNA expression. The positive rate of DKK2 mRNA expression in cervical cancer decreased significantly compared to that in HSIL (χ^2^ = 5.999, *P* = 0.014). The positive rate of DKK2 mRNA expression in HSIL was significantly lower than that in normal cervical samples (χ^2^ = 17.385, *P* = 0.00003), and LSIL (χ^2^ = 15.318, *P* = 0.001). The RT-PCR results for the 2 normal cervical tissues (N1, N2), 2 LSIL (L1, L2), 2 HSIL (H1, H2), and 11 primary tumors (T1 to T11) analyzed are shown in **Figure 1H**.

**Table 2:** *DKK2* mRNA expression and methylation in cervical neoplasms

**Diagnosis**	**n**	**mRNA expression (%)**	***P***	**Methylation (%)**	***P***
Normal	29	29/29 (100)		0/29 (0)	
LSIL	25	25/25 (100)	0.00003	1/25 (4.0)	0.005
HSIL	38	21/38 (55.2)	0.001	9/38 (23.7)	0.037
Cervical cancer	79	25/79 (31.6)	0.014	41/79 (51.9)	0.004

DKK2, Dickkopf 2; HSIL, high-grade squamous intraepithelial lesions; LSIL, low-grade squamous intraepithelial lesions.

### Correlation of *DKK2* inactivation with its promoter hypermethylation

**Figure 2:**
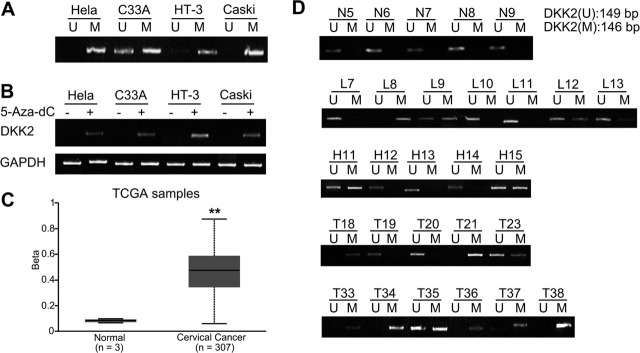
*DKK2* methylation level in cervical cancer cell lines and tissues. (**A**) MSP analysis of *DKK2* hypermethylation in Hela, Caski, HT-3, and C33A cervical cancer cell lines. (**B**) *DKK2* expression after treatment with 5-Aza-dC for 4 d in cervical cancer cell lines. GAPDH was used as a housekeeping control gene. (**C**) *DKK2* methylation levels in TCGA samples analyzed by data mining. ^**^P < 0.01 compared with normal tissues. (**D**) MSP analysis of *DKK2* CGIs methylation frequency in primary cervical cancer tissues. CGIs, CpG islands; DKK2, Dickkopf 2; GAPDH, glyceraldehyde-3-phosphate dehydrogenase; M or MSP, methylation-specific polymerase chain reaction (PCR); TCGA, The Cancer Genome Atlas; U, unmethylated-specific PCR.

We then examined the status of *DKK2* promoter methylation in HeLa, HT-3, C33A, and CaSki cell lines by MSP. *DKK2* hypermethylation was detected in all 4 cell lines (**Figure 2A**). Of these 4 cell lines, the C33A cell line showed the weakest DKK2 mRNA expression, although both unmethylated and methylated bands were detected. Correlation analysis between *DKK2* silencing and promoter hypermethylation was performed by treating cell lines with 10 μM 5-Aza-dC induction for 4 d, followed by promoter methylation level analysis. DNA-demethylating agent treatment significantly led to *DKK2* demethylation and re-expression of the transcript among all 4 cell lines analyzed (**Figure 2B**).

We then examined *DKK2* methylation status in cervical tissues. Methylation levels were significantly higher in cervical cancer tissues, as shown in TCGA samples (**Figure 2C**, *P* < 0.0001). MSP analysis was then performed to confirm the overall frequency of *DKK2* methylation in cervical cancer. *DKK2* promoter hypermethylation was detected in 41 of 79 cases (51.9%) of cervical cancer tissues. However, methylation status was only found in 10 of 63 CIN cases (12.7%), and no methylation status was detected in normal cervical cases (**Figure 2D**). The positive rate of *DKK2* methylation in cervical cancer was significantly higher than that in HSIL (χ^2^ = 8.346, *P* = 0.004), whereas the positive rate of *DKK2* methylation in HSIL was significantly higher than that in normal cervical samples (χ^2^ = 7.934, *P* = 0.005) and in LSIL samples (χ^2^ = 4.375, *P* = 0.037) (**Table 2**). Positive DKK2 mRNA expression in cervical cancer cases without methylation (47.4%) was significantly higher than that of cervical cancer tissues with promoter hypermethylation (17.1%) (χ^2^ = 8.368, *P* = 0.002).

### Clinicopathological importance of *DKK2* promoter hypermethylation

The correlation between *DKK2* hypermethylation and the primary clinicopathological characteristics of cervical cancer, including histology, stage, lymph node metastasis, HPV infection, and differentiation, was investigated further. *DKK2* methylation (76.5% vs 45.2%, respectively, χ^2^ = 5.239, *P* = 0.022) and DKK2 mRNA expression (11.8% vs 37.1%, respectively, χ^2^ = 3.958, *P* = 0.047) are significantly different between patients with or without lymph node metastasis (**Table 3**). The rate of detection of *DKK2* methylation in HPV16/18-positive patients in the cervical cancer group (66.7%) was significantly higher than that in HPV16/18-negative patients (36.0%) (χ^2^ = 6.279, *P* = 0.015). The rate of detection of *DKK2* methylation in HPV16/18-positive patients (24.1%) in the precancerous lesion group was slightly higher than that in HPV16/18-negative patients (13.0%) (χ^2^ = 1.016, *P* = 0.482) (**Table 4**). No significant association was detected between *DKK2* hypermethylation and other cervical cancer clinicopathological characteristics, such as clinical stage (*P* = 0.32), histology (*P* = 0.55), and tumor grade (*P* = 0.17) (**Table 3**).

**Table 3:** DKK2 mRNA expression, *DKK2* promoter methylation and clinicopathological characteristics of cervical cancer

**Clinical status**	**n**	**DKK2 mRNA expression**			***DKK2* methylation**		

		**+**		***P***	**+**		***P***
Clinical stage							
Stage IA2	28	13 (46.4%)	15 (53.6%)	0.099	12 (42.9%)	16 (57.1%)	0.32
Stage IB1+IB2	41	9 (22.0%)	32 (78.0%)		22 (53.7%)	19 (46.3%)	
Stage IIA	10	3 (30.0%)	7 (70.0%)		7 (70.0%)	3 (30.0%)	
Histology							
Squamous cell carcinoma	70	21 (30.0%)	49 (70.0%)	0.38	36 (51.4%)	34 (48.6%)	0.55
Adenocarcinoma	9	4 (44.4%)	5 (55.6%)		5 (55.6%)	4 (44.4%)	
Tumor grade							
Grade 1	15	5 (33.3%)	10 (66.7%)	0.81	6 (40.0%)	9 (60.0%)	0.17
Grade 2	42	12 (28.6%)	30 (71.4%)		20 (47.6%)	22 (52.4%)	
Grade 3	22	8 (36.4%)	14 (63.6%)		15 (68.2%)	7 (31.8%)	
Lymph node metastasis							
Negative	62	23 (37.1%)	39 (62.9%)	0.047	28 (45.2%)	34 (54.8%)	0.022
Positive	17	2 (11.8%)	15 (88.2%)		13 (76.5%)	4 (23.5%)	

DDK2, Dickkopf 2.

**Table 4:** Relationship between HR-HPV infection type and DKK2 methylation rate

**Group**	**HR-HPV (+)** **n**	***DKK2* methylation**		***P***

		**n**	**%**	
Cervical cancer				0.015
HPV16/18(+)	48	32	66.7	
HPV16/18(−)	25	9	36	
Precancerous lesions				0.48
HPV16/18(+)	29	7	24.1	
HPV16/18(−)	23	3	15	

HPV, human papillomavirus; HR-HPV, high-risk HPV.

## Discussion

In the present study, we investigated *DKK2* expression profiles and epigenetic alterations in cervical cancer. DKK2 mRNA expression was reduced in cell lines of cervical cancer and cancer tissues, whereas *DKK2* hypermethylation was upregulated. In addition, DKK2 mRNA expression was restored after 5-Aza-dC treatment of cell lines of cervical cancer. The study suggests *DKK2* silencing is strongly associated with its promoter hypermethylation.

The activated Wnt/β-catenin signaling pathway plays an important role in cervical cancer. CpG island promoter hypermethylation has been shown to inactivate extracellular Wnt antagonists in cervical cancer [22, 23]. CpG island methylation can be widely found in the human genome, but only a subset of loci plays important roles in tumorigenesis. These implicated genes are known to be involved in multiple cellular signaling pathways such as apoptosis, cell cycle regulation, development, differentiation, invasion, and metastasis. As CpG island methylation occurs early and methylated alleles can be detected in a sensitive manner in carcinogenesis, detection of methylation may be a promising tool for early detection of cancer [24]. DKK2, a Wnt antagonist, contributes to tumorigenesis in multiple cancers [12, 13, 25,26,27,28]. In the present study, RT-PCR showed reduced expression of DKK2 mRNA in cervical cancer compared with either normal cervical samples or HSIL samples. Further, through MSP analysis *DKK2* is found to be methylated in most patients diagnosed with cervical cancer, while it is not in HSIL and normal cervical samples. Collectively, the data demonstrate that *DKK2* is predominantly methylated in cervical cancer. *DKK2* methylation status and gene expression level showed a significant inverse correlation.

Methylation of the *DKK1* promoter in patients with cervical squamous cell carcinoma is related to high-risk HPV infection and histological differentiation, tumor size, lymph node metastasis, and International Federation of Gynecology and Obstetrics (FIGO) staging, while the degree of methylation of *DKK1* is not related to the type of high-risk HPV infection [14]. Paired *PAX1* methylation was found to be a valuable biomarker for cervical cancer screening, a commonly used method in our hospital, with a 77% sensitivity and 92% specificity of CIN3+ versus normal [29]. A high methylation rate of *DKK2* was significantly associated with poor overall survival, and a multivariate Cox proportional hazards model revealed that methylation of *DKK2* is an independent adverse prognostic factor [28]. In the present study, significant *DKK2* hypermethylation was detected in lymph node-positive cervical cancer. The methylation rate of the *DKK2* promoter was 76.5% in cervical cancer specimens with lymph node metastasis, and only 45.2% in cervical cancer specimens without lymph node metastasis, indicating a significant difference between the 2 groups.

HPV infection accounts for over 90% of cervical cancer cases, and the high-risk types of HPV are associated with 87%–88% of squamous cell carcinomas [30]. A triage test using DNA methylation may be helpful to differentiate women who are at high risk of developing cervical cancer rapidly from women with low risk. In the case of HSIL, the combination of HPV genotyping and methylation marker analysis can not only overcome the limitations of cytological examination but also increase diagnosis accuracy. The present study indicated that among HR-HPV-positive cervical cancer patients, the methylation rate of *DKK2* in HPV16/18-positive patients was more greatly enhanced than that in HPV16/18-negative patients.

We acknowledge that the present study also has some limitations. In the precancerous lesion group, the difference was not statistically significant. The correlation between *DKK2* methylation level and HPV subtype needs to be investigated further by including a larger sample size. In addition, the population included in the present study was drawn from patients in hospitals, not from a population that would be screened. The real-world screening value of *DKK2* methylation detection for cervical cancer needs further population-based research and discussion.

## Conclusion

*DKK2* epigenetic changes may play a key role in the development of cervical cancer, suggesting a potential value of *DKK2* hypermethylation as a triage test for screening, early diagnosis, or predicting the risk of cervical cancer.
